# An Unusual Presentation of Diffuse Large B-Cell Lymphoma

**DOI:** 10.7759/cureus.20927

**Published:** 2022-01-04

**Authors:** Aikaterini Gkoufa, Vasiliki E Georgakopoulou, Eleftheria Lakiotaki, Evangelos Cholongitas

**Affiliations:** 1 First Department of Internal Medicine, Laiko General Hospital, School of Medicine, National and Kapodistrian University of Athens, Athens, GRC; 2 Department of Pulmonology, Laiko General Hospital, Athens, GRC; 3 First Department of Pulmonology, Sismanogleio Hospital, Athens, GRC; 4 First Department of Pathology, School of Medicine, National and Kapodistrian University of Athens, Athens, GRC

**Keywords:** liver infiltration, diffuse large b-cell lymphoma, liver biopsy, liver lymphoma, lactic acidosis

## Abstract

Hyperlactemia is a rare and potentially fatal complication of hematologic malignancies, as well as an oncological emergency, which requires a fast diagnosis and early therapeutic management, as these interventions may alter disease prognosis. Herein, we present a case of secondary liver-biopsy-confirmed diffuse large B-cell lymphoma (DLBCL), presented with elevated liver enzymes and lactic acidosis, without depicted hepatic lesions, hepatosplenomegaly, or enlarged lymph nodes on computed tomography. This case confirms the poor prognosis of cases with delayed diagnostic intervention and highlights the importance of early clinical suspicion, liver biopsy, and prompt treatment initiation.

## Introduction

Diffuse large B-cell lymphoma (DLBCL) is the most common B-cell non-Hodgkin lymphoma (NHL) in adults and secondary involvement of the liver is relatively common in the course of the disease, occurring in 16-46% of patients [1,2]. Any unusual but urgent manifestation related to DLBCL, such as type B lactic acidosis, hypoglycemia, and renal infiltration of acute liver failure [1,3,4], should raise the suspicion of the clinician to make a prompt diagnosis, especially in cases where rapid treatment commencement may alter disease prognosis. Type B lactic acidosis, occurring under adequate tissue perfusion, as opposed to type A, where tissue hypoxia is present, is a rare and potentially fatal complication of hematologic malignancies [5]. It is also associated with poor prognosis and included in oncological emergencies [5]. The mechanism underlying its pathogenesis may be multifactorial and may portend severe hepatic or renal dysfunction, where lactate metabolism is impaired, thus leading to its accumulation [6]. Other possible mechanisms involve enhanced aerobic glycolytic activity in tumor cells, a process called the "Warburg effect," which is the overexpression of certain glycolytic enzymes [6]. This process may simultaneously cause hypoglycemia or a deficiency of thiamine or riboflavin [7].

There are reports of NHL cases, either presented initially or during disease progression or recurrence, with lactic acidosis with or without hypoglycemia and with or without liver involvement [3,4,6-14]. All these reports indicate a high mortality rate, and any treatment delay may further contribute to devastating outcomes [3,4,6-14]. Herein, we report a patient who presented with fever, elevated liver enzymes, and type B lactic acidosis, diagnosed with DLBCL after a liver biopsy.

## Case presentation

A 72-year-old Caucasian woman with a past medical history of hypercholesterolemia, treated with pitavastatin, presented to our department due to a two-month history of low-grade fever, weakness, and night sweats. On admission, the patient was febrile and normotensive, while the physical examination was unremarkable. Laboratory analysis showed normochromic, normocytic anemia with elevated C-reactive protein (CRP), erythrocyte sedimentation rate (ESR), lactate dehydrogenase (LDH), and mildly elevated liver enzymes (Table 1).

**Table 1:** Laboratory investigations of the patient on admission.

Serum parameters	Patient’s data	Reference range
White blood cells	6,8 x 10^3^/μL	4-10 x 10^3^/μL
Neutrophils	4,7 x 10^3^/μL	1.5-6.6 x 10^3^/μL
Platelets	155.000/μL	140-440.000/μL
Hemoglobin	8.8 g/dl	12-16 g/dl
C-reactive protein	235 mg/L	0-5 mg/L
Erythrocyte sedimentation rate	92 mm/h	0-20 mm/h
Lactate dehydrogenase	895 U/L	135-214 U/L
Creatinine	0.9 mg/dl	0.6-1 mg/dl
Aspartate transaminase	95 U/L	13-35 U/L
Alanine transaminase	77 U/L	13-33 U/L
Gamma-glutamyl transferase	46 U/L	5-36 U/L
Alkaline phosphatase	140 U/L	35-104 U/L
Total bilirubin	0.90 mg/dL	0.3-1.2 mg/dL

Multiple blood tests, urine cultures, and a purified protein derivative (PPD) test were negative. Viral serology tests, including hepatitis A virus (HAV), hepatitis B virus (HBV), hepatitis C virus (HCV), Epstein-Barr virus (EBV), cytomegalovirus (CMV), and human immunodeficiency virus (HIV), as possible causes for increased liver function tests, were not indicative of a recent or chronic infection. Laboratory studies with antinuclear antibodies (ANA), antimitochondrial antibodies (AMA), anti-liver-kidney microsomal antibodies, and anti-soluble liver antigens were negative. A peripheral blood smear was evaluated and did not show any abnormal cells.

**Figure 1:**
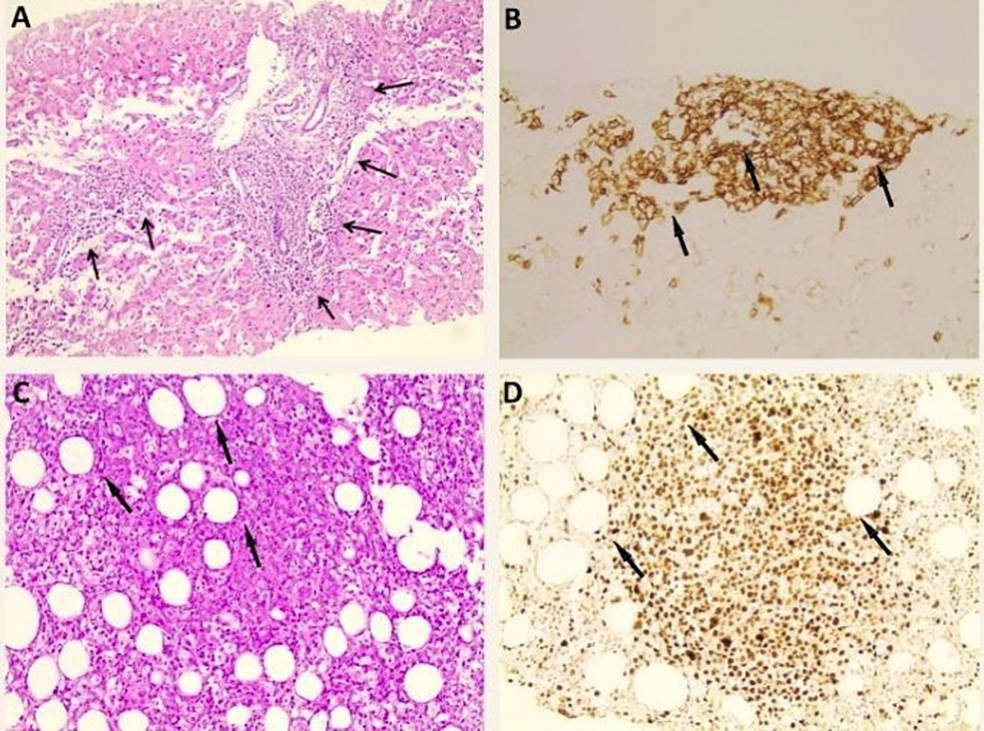
Pathology findings from liver and bone marrow biopsy. A-B: Pathology findings from liver biopsy. Arrows show extended portal tracts due to diffuse infiltration by large-sized lymphoid cells, with a minimal sinusoidal pattern. The lymphoid cells were positive for the B cell marker CD20 and negative for the T cell marker CD3. C-D: Pathology findings from bone marrow biopsy. Arrows show diffuse extensive infiltration by large lymphoid cells that were positive for the markers CD20, CD5, MUM1, c-myc, Bcl-2, and Bcl-6, and negative for CD3, CD10, CD30, and cyclin D1, with a very high proliferation index Ki67 (90%), consistent with CD5+ DLBCL of non-germinal center (non-GC) subtype. DLBCL: diffuse large B-cell lymphoma; MUM1: multiple myeloma oncogene-1; Bcl-2: B-cell lymphoma 2; Bcl-6: B-cell lymphoma 6.

Computed tomography (CT) of the brain, chest, and abdomen did not reveal any findings. On the fourth day of hospitalization, and as the patient continued to be febrile, thus meeting the criteria for a fever of unknown origin, a venous blood gas revealed an elevated lactate level (8 mmol/L), pH (7.39), and bicarbonate (HCO3) (20.3 mmol/L). Sepsis, cardiogenic shock, and hypoperfusion were excluded due to the patient’s normal blood pressure and newly obtained sterile blood and urine culture. Further evaluation with gastrointestinal endoscopies was unremarkable. Due to the marked rise of liver enzymes, lactate level, and LDH, we decided to perform a liver and bone marrow biopsy. Bone marrow aspiration was not diagnostic. The bone marrow biopsy results were delayed, while liver biopsy diagnosed DLBCL (Figures 1A, 1B). A few days later, and while the patient continued to deteriorate with a lactate level of 10.6 mmol/L and pH of 7.33, a bone marrow biopsy confirmed DLBCL (Figures 1C, 1D). Soon after diagnosis, and before any treatment initiation, the patient died due to severe respiratory failure arising from hospital-acquired pneumonia after a prolonged hospital course.

## Discussion

Liver biopsy is the mainstay in the diagnosis of primary hepatic lymphoma, while secondary NHLs are usually diagnosed after a lymph node biopsy, or in rarer cases, after bone marrow aspiration and biopsy [15]. In the literature, there are only three cases of secondary hepatic NHL, presented with lactic acidosis and diagnosed with liver biopsy [10,16]. Table 2 summarizes the cases of lymphoma presented with lactic acidosis and diagnosed with liver biopsy.

**Table 2:** Cases of lymphoma presented with lactic acidosis and diagnosed with liver biopsy. DLBCL: diffuse large B-cell lymphoma.

Case	Age/sex	Imaging findings	Diagnosis based on liver biopsy	Outcome
Kestler et al. (2010) [10]	40 years/male	Hepatomegaly with multiple lesions, lymphadenopathy	Burkitt lymphoma	Death seven months after diagnosis
Kestler et al. (2010) [10]	48 years/male	Hepatomegaly with multiple lesions	DLBCL	Death soon after presentation
Keller et al. (2010) [16]	53 years/male	Hepatomegaly, splenomegaly, lymphadenopathy	DLBCL	Treatment response
Our case	72 years/female	Normal	DLBCL	Death at initial presentation

Kestler et al. reported a patient with a recent diagnosis of HIV infection, who presented with night sweats, dysphagia, and postprandial abdominal pain and elevated LDH, lactate level, and liver enzymes [10]. His abdominal CT revealed hepatomegaly with multiple lesions and associated bulky adenopathy of the gastroesophageal junction, while the liver biopsy showed infiltration of DLBCL [10]. In the same report, Kestler et al. reported a second patient with a prior history of HIV infection, who presented with dyspnea, abdominal discomfort, and hyperlactatemia [10]. Due to significant hepatomegaly and elevated liver enzymes, the performance of liver biopsy was decided; however, the patient died after multi-organ system failure and a post-mortem liver biopsy revealed DLBCL [10]. Keller et al. reported a patient with fever, nausea, abdominal discomfort, diarrhea, elevated lactate level, hypoglycemia, and increased liver function tests, with hepatomegaly, splenomegaly, and abdominal lymphadenopathy. The patient’s liver biopsy demonstrated a DLBCL [16]. All patients had image confirmation of liver involvement [16].

To the best of our knowledge, this is the first reported case of DLBCL presented with laboratory findings of lactic acidosis and elevated liver enzymes, and diagnosed with liver biopsy, without depicting hepatic lesions, hepatomegaly, or enlarged lymph nodes on abdominal CT. Imaging features of secondary hepatic lymphoma may range from diffuse infiltration to multifocal homogenous lesions or miliary patterns, with extrahepatic involvement (splenic lesions, lymphadenopathy, and bone marrow infiltration) [17]. Despite relatively common liver involvement in NHLs, in rare cases, liver and bone marrow may be the only sites of the disease, thus complicating diagnosis, especially in cases where hepatomegaly or focal hepatic lesions are absent [18]. In such cases, and as liver involvement is considered as evidence of advanced disease and the diffuse infiltration of the liver has no specific imaging features, any abnormality of liver enzymes should be promptly evaluated and a liver biopsy should be obtained as early as possible [18]. In our patient, fluorodeoxyglucose positron emission tomography-computed tomography (FDG-PET/CT) was potentially helpful for biopsy guidance; however, this diagnostic test was not available in our hospital and the patient’s clinical deterioration during hospitalization was a deterrent factor for her referral. Concerning lactic acidosis, after the exclusion of hypoperfusion, it was finally attributed both to liver involvement and the metabolic effects of the lymphoma. Although bicarbonate and thiamine administration, as well as hemodialysis, have been used for the management of type B lactic acidosis, the only effective management focuses on the treatment of underlying malignancy and identification [12,13,19]. The inciting etiology constitutes the main challenge for clinicians [19].

## Conclusions

Type B lactic acidosis, an uncommon and challenging presenting feature of hematologic malignancies, is associated with poor prognosis and highlights that timely detection of the underlying disease is crucial for patients’ improvement and survival. Unexplained lactic acidosis in a patient with increased liver function tests, even in cases without focal hepatic lesions or hepatomegaly, should raise strong suspicions and guide the clinician in performing a liver biopsy without delay. Most hematologists are quite familiar with this manifestation in patients with an already known hematologic malignancy; however, an undiagnosed patient, hospitalized in an internal medicine department, requires the vigilance of experienced physicians and clinicians to provide an efficient evaluation, quick diagnosis, and early therapeutic management.
